# Effects of fat source in calf starter on growth performance, blood fatty acid profiles, and inflammatory markers during cold season

**DOI:** 10.1038/s41598-023-45956-w

**Published:** 2023-10-30

**Authors:** H. Khalilvandi-Behroozyar, B. Mohtashami, M. Dehghan-Banadaky, M. Kazemi-Bonchenari, M. H. Ghaffari

**Affiliations:** 1 Department of Animal Science, Urmia University, Urmia, 5756151818 Iran; 2 Department of Animal Science, University of Tehran, Tehran, 3158777871 Iran; 3 Department of Animal Science, Faculty of Agriculture and Natural Resources, Arak University, Arak, 38156-8-8349 Iran; 4 Institute of Animal Science, University of Bonn, 53111 Bonn, Germany

**Keywords:** Developmental biology, Physiology

## Abstract

This study was conducted to investigate the effects of supplementation of different fat sources in calf starters on growth performance, health, blood fatty acid profiles, and inflammatory markers during the cold season in dairy calves. A total of 48 Holstein calves (24 males and 24 females) were randomly assigned to 1 of 4 starter diets throughout the experiment (d 3 to 65): (1) no supplemented fat (CON), (2) 3% calcium-salts of soybean oil (Ca-SBO), (3) 3% calcium-salts of fish oil (Ca–FO), and (4) 3% mixture of Ca-SBO and Ca–FO (1.5% each, DM basis; MIX). Calves were given free access to starter feed and water and were raised individually in pens from 3 to 65 d of age. Calves fed Ca-SBO consumed a greater proportion of n-6 FA, while calves fed Ca–FO consumed a greater level of n-3 FA compared to the other dietary treatments. Fat supplementation increased the intake of linoleic acid, the major n-6 FA, with the greater intake observed in the Ca-SBO group compared to the other dietary treatments. Calves fed the Ca–FO and MIX diets consumed more long-chain n-3 FA than the other diets. In addition, calves fed Ca-SBO and Ca–FO diets consumed more starter feed and total dry matter than calves fed MIX and CON throughout the experiment (d 3 to 65). Calves fed Ca–FO had higher average daily gain throughout the trial (d 3 to 65) than the other treatment groups. Of all treatment groups, calves fed Ca–FO achieved the highest final body weight and showed the greatest feed efficiency. Random forest analysis revealed that eicosapentaenoic acid (EPA), docosahexaenoic acid (DHA), and arachidonic acid were the serum levels of FA most affected by the diets. The principal component analysis of blood FA profile, blood parameters, and inflammatory markers showed distinct differences between dietary treatments. Calves fed Ca-SBO had higher plasma concentrations of linoleic acid, while calves fed Ca–FO had higher plasma concentrations of long-chain n-3 polyunsaturated fatty acids (PUFA), such as EPA, docosapentaenoic acid (DPA), and DHA than the other treatment groups. Plasma inflammatory markers were lower in calves fed Ca–FO and higher in calves fed CON than in the other treatment groups. The Ca–FO group had lower levels of inflammatory markers, including serum amyloid A, tumor necrosis factor-alpha, Interferon-γ, haptoglobin, and interleukin-6 compared to the other experimental treatments. Also, the blood malondialdehyde levels, an indicator of oxidative stress, were lower in calves fed Ca–FO compared with calves fed the other treatment diets. In conclusion, the performance of preweaned dairy calves can be improved by adding fat to their starter feed under cold conditions. Overall, the type of fat in milk may affect growth and inflammation of dairy calves before weaning under cold conditions, with n-3 FA (Ca–FO) promoting growth and reducing inflammation more effectively than n-6 FA (Ca-SBO).

## Introduction

Calves are born with limited body fat stores, and even at 6 weeks old, their body fat percentage remains below 4%^1^. Calves can respond to low ambient temperatures with thermogenesis, but their body temperature regulation is not as mature as older animals, so cold ambient temperatures are detrimental to their health and survival^2–4^. Calves under cold conditions increase starter feed intake as a biological strategy to provide needed energy^5^. Previous studies found that calves raised in cold weather consumed more starter feed than calves born in other seasons but had the same weight gain^5^. These results suggest that this biological strategy cannot provide optimal weight gain for calves raised under cold stress.

Dairy calves were fed supplemental fat to increase their energy intake^6^. Under mild cold stress conditions, supplementation of milk replacers with fat resulted in greater weight gain at an early age^7^. Adding fat to the diet of dairy calves has produced conflicting results, with some studies reporting negative effects^8^, and others reporting positive results^9^. These discrepancies in calf responses to supplemental fat may be due to several factors, including the type of fat used^6,8^, the amount of fat added^10^, and the method of administration^11^. Additionally, environmental conditions also play a crucial role in the response of animals to added fat^4,12,13^. Ghasemi et al.^12^ found that of the different fat sources fed to dairy calves during the cold season (palm fat, soybean oil, tallow, and a mixture of three sources), only soybean oil supplementation improved animal growth performance. Furthermore, previous studies have shown that some individual FA can improve animal immune function^9,14^.

Although few studies have examined the effects of fat sources in calf starters during cold stress, previous research has not considered the effects of partially protected sources such as calcium salts on calf immune response and inflammatory markers under cold conditions. There is a significant lack of knowledge on whether fat supplementation improves animal growth performance by increasing dietary energy content or regulating inflammatory markers via FA supplementation. It was hypothesized that a starter feed supplemented with fat during the cold season would improve the growth performance of preweaned calves compared to non-supplemented calves. Furthermore, feeding fish oil n-3 FA was hypothesized to result in lower inflammatory markers than calcium salts and soybean oil n-6 FA. Therefore, this study aims to evaluate the effects of calcium salts from soybean oil and fish oil, alone and in combination, on intake, growth performance, health, blood parameters, and inflammatory markers in Holstein dairy calves during the cold season.

## Materials and methods

### Weather conditions

The experiment was conducted on a commercial dairy farm (approximately 15,000 lactating dairy cattle) in Moghan Agriculture-Industrial and Livestock Co. (Parsabad Moghan, Iran) from December 2018 to February 2019, the typical cold season in the region. Table 1 shows that the average monthly temperature values for December, January, and February at the experimental site were − 2.6 °C, − 3.0 °C, and − 1.7 °C, respectively. The lowest and highest temperatures were recorded in January (− 3.0 °C) and December (11.3 °C), respectively. The highest and lowest monthly humidity were 88.9% and 54.6%, respectively. Animal Care and Use Committee of Urmia University approved all animal experiments (ACUC Protocol #IR2018011) according to the Iranian Council of Animal Care^15^. The study complies with ARRIVE guidelines for reporting in vivo experiments, and all methods were performed following the relevant guidelines and regulations. Experimental research and field studies on plants (cultivated or wild), including the collection of plant material, must comply with relevant institutional, national, and international guidelines and legislation.

**Table 1:** Minimum and maximum temperature and humidity by month throughout cold season.

Month	Temperature (ºC)			Humidity (%)		Wind velocity (km/h)
	Max	Min	Mean	Max	Min	
December 2018	11.3	− 2.6	2.53	88.2	54.6	6.62
January 2019	8.0	− 3.0	1.98	88.3	59.2	8.30
February 2019	8.3	− 1.7	3.51	88.9	58.8	10.3

### Calves and experimental design

Forty-eight newborn Holstein calves (12 calves/treatment; 6 males and 6 females) with an average age of 3 ± 1 day and a body weight (BW) of 41.8 ± 3.53 kg were assigned in a completely randomized design trial. All calves were assigned to experimental treatments on the same day, and the trial was started on a constant day for all treatments. Calves were raised in individual pens (2.2 m length × 1.1 m width) from 3 to 65 days of age throughout the experiment. Calves received 6 L of colostrum within the first 12 h of life (3 L of colostrum within 2 h of life and 3 L at a second feeding). Colostrum quality was measured using a digital refractometer (Digital dairy model, DD-3, Misco, Cleveland, OH, USA). The colostrum used in the experimental calves exhibited high quality, as evidenced by its Brix index of 25.25 ± 2.54%, total solids content of 27.32 ± 2.53%, and pH of 6.31 ± 0.08. Consequently, the calves had an average serum total protein content of 5.71 ± 0.39 mg/dL on 48 ± 4 h after birth.

Milk was prepared twice daily (at 08:00 and 17:00 h) in steel pails according to the following schedule: 4 L/d from d 3 to 30, 6 L/d from d 31 to 50, 4 L/d from d 51 to 60, and 2 L/d of milk (once daily) during morning feeding from d 61 to 64 of age. Whole milk (38 °C) was pasteurized and routinely analyzed with a milk scanner (MilkoscanTMS50-75610, FOSS Nils Foss Allé 1, DK-3400 Hilleroed, Denmark). The data were used to calculate the DM intake of the calves. The average composition of the milk was 3.3% protein, 3.5% fat, 4.2% lactose, and 13% of total solids.

The experimental treatments were as follows: (1) Starter diet without fat supplementation (CON), (2) 3% Calcium salts of soybean oil (Ca-SBO), (3) 3% Calcium salts of fish oil (Ca–FO), (4) 3% Mixture of SBO and FO (1.5% each, base DM: MIX), Calcium salts of SBO and Calcium salts of FO were provided by Kimiya Danesh Alvand CO (Persia Fat®, Tehran, Iran) as described in Table 2. The ingredients and composition of the experimental diets are described in Table 3. To avoid possible oxidation of FA due to the long storage time, starters were prepared in small batches and at short intervals during the experimental period.

**Table 2:** Chemical composition and fatty acid profiles of calcium salts of soybean oil and calcium salts of fish oil used in this study.

Items	Ca-salts of soybean oil^1^	Ca-salts of fish oil^1^
Dry matter (g/kg)	97.11 ± 1.13	97 ± 1.41
Ash (g/kg DM)	13.12 ± 1.15	14.57 ± 1.12
Ca (g/kg DM)	11.24 ± 0.57	12.02 ± 0.47
NE_l_ (MJ/kg DM) ^b^	5.8	5.8
Total fat (g/kg DM)	85.34 ± 1.34	84.67 ± 1.05
Fatty acid profiles (g/kg Fat)		
C16:0	15.11 ± 1.06	19.63 ± 0.11
C16:1	2.57 ± 0.24	3.38 ± 0.18
C18:0	5.47 ± 0.11	5.67 ± 0.09
C18:1	24.06 ± 1.44	28.21 ± 0.67
C18:2	48.12 ± 2.11	25.11 ± 1.11
C18:3	2.37 ± 0.31	2.12 ± 0.06
C20:5	ND	6.11 ± 0.24
C22:5	ND	1.24 ± 0.03
C22:6	ND	8.27 ± 0.21

Chemical composition and fatty acid profiles were determined in 3 replicates.

ND = not detected.

^1^ Provided by the manufacturer based on digestibility studies and calculations based on NRC (2001) equations.

^1^ Ca-salts of fatty acids were provided by Kimiya Danesh Alvand Co.

**Table 3:** Ingredient and chemical composition of starter and mixed diets in experimental treatments.

Item	Treatment ^a^			
	CON	Ca-SBO	Ca–FO	Mix
Ingredients, %				
Alfalfa hay	10	10	10	10
Corn grain, cracked	46	40.5	40.5	40.5
Barley grain, cracked	13.5	13.5	13.5	13.5
Soybean meal	26.1	26.5	26.5	26.5
Wheat bran	1.0	4.2	4.2	4.2
Omega-6 Ca-salts^b^	–	2.7	–	1.35
Omega-3 Ca-salts^b^	–	–	2.7	1.35
Di–Ca Phosphate	0.36	0.36	0.36	0.36
Calcium carbonate	1.09	0.9	0.9	0.9
Sodium bicarbonate	0.72	0.72	0.72	0.72
Vitamins and minerals premix^c^	0.45	0.45	0.45	0.45
White salt	0.18	0.18	0.18	0.18
Chemical composition^d^ DM basis				
ME, Mcal/kg^e^	3.10	3.12	3.12	3.12
CP (%)	19.7	19.7	19.6	19.7
Ether Extract (%)	3.0	5.6	5.6	5.6
NDF (%)	18	17.9	17.9	17.9
ADF (%)	10	10.1	10.0	10.1
Ash (%)	7.6	7.7	7.7	7.7
Ca (%)	0.15	0.18	0.18	0.18
P (%)	0.58	0.57	0.57	0.58
Fatty acids, %				
C16:0	13.8	14.3	17.1	15.8
C16:1	0.2	1.0	1.5	1.3
C18:0	2.7	2.8	4.0	4.1
C18:1	25.7	26.0	25.5	25.6
C18:2	52.8	52.7	40.4	45.0
C18:3	3.0	2.8	2.7	2.9
EPA, DPA, DHA ^f^	ND	ND	7.4	3.6
Others	1.8	0.4	1.4	1.7
Saturated fatty acids	17.1	17.2	21.7	20.4
Unsaturated fatty acids	82.9	82.8	78.3	79.6

^a^Treatments were: CON: no supplemented fat source, Ca-SBO: calcium slats of soybean oil (3%, DM basis), Ca–FO: calcium slats of fish oil (3%, DM basis); MIX: a mixture of Ca-SBO and Ca–FO (1.5% each, DM basis).

^b^The Ca-Salts of soybean oil and Ca-Salts of fish oil (Persia-Fat) were provided by Kimiya Danesh Alvand Co. (Tehran, Iran).

^c^Mineral-vitamin pre-mix contains: 250,000 IU of vitamin A, 50,000 IU of vitamin D, 1,500 IU of vitamin E, 120 g of Ca, 20 g of P, 20.5 g of Mg, 186 g of Na, 7.7 g of Zn, 2.25 g of Mn, 1.25 g of Fe, 3 g of S, 14 mg of Co, 1.25 g of Cu, 56 mg of I, and 10 mg of Se.

^d^Chemical composition and fatty acid profiles of the starter and mix diets were laboratory analyzed in 3 replicates.

^e^Calculations based on NRC (2001) equations.

^f^Eicosapantanoic acid (EPA; 20:5n-3); Docosapentaenoic acid (DPA; 22:5n-3); Docosahexaenoic acid (DHA; 22:6n-3).

### Intake measurement and health indicators recording

Daily measurement of feed intake was done using a calibrated digital scale with a maximum weight capacity of 6 kg ± 0.1 g at 100 mg readability and a minimum piece weight of 5 g (Kern and Sohn, model Kern EW 6000-1 M, Stuttgart, Germany). Body weight was taken using a calibrated digital scale with a maximum capacity of 500 kg ± 100 g (1.5 m length × 1 width, Pand Electronics, Tehran, Iran) every two weeks. The content of feed offered and refused was recorded daily for each calf. Calves were fed textured starters ad libitum to permit at least 10% orts. The ADG (kg of BW/d) was calculated at weighing intervals, and feed efficiency was determined as kg of ADG/total DM intake (DMI; liquid feed DMI + starter feed DMI). Measurements of skeletal growth, including body length, body girth, withers height, hip and pin width, and pin-to-hip width, were done at d 3, 30, and 60 using the method described previously^16^. Weekly samples of calf starters offered were analyzed for dry matter (No. 934.01), ether extract (No. 954.02), and crude protein (No. 988.05) according to AOAC^17^. Dietary fiber contents (ADF and NDF) were analyzed following Van Soest et al.^18^. Chopped alfalfa hay was added to the calf starter diets (10%, DM basis) initiated from d 21. Systematic weekly assessments showed that feed refusals contained an average of 12 ± 3% forage particles.

Rectum and ear skin temperatures were measured and recorded using a digital and infrared thermometer^19^ (ZOTEK- GM320, Zotek tools, Shenzhen, China). The health scores of the calves were recorded daily according to the calf health chart^20^. The respiratory clinical score was calculated according to McGuirk and Peek^21^. Fecal scores were established as (0) normal, (1) semi-formed, pasty, (2) loose, but stays on top of bedding, (3) watery, sifts through bedding. Ear scores: (0) normal, (1) ear flick or head shake, (2) slight unilateral droop, (3) head tilt or bilateral droop. Eye scores: (0) normal, (1) small amount of ocular discharge, (2) moderate amount of bilateral discharge, (3) heavy ocular discharge. Nasal scores: (0) normal serous discharge, (1) small amount of unilateral cloudy discharge, (2) bilateral, cloudy or excessive mucus discharge, (3) copious bilateral mucopurulent discharge. All health incidents, and treatments were recorded for the length of the study.

Fecal samples from healthy calves were collected through rectal palpation into sterile containers on d 58 to 60, before morning milk feeding to count the colony number of major beneficial and pathogenic bacterial species. The pour plate method and MacConkey agar (HiMedia Laboratories Pvt. Limited, India) were used for counting *E. coli* and *Salmonella*. Moreover, MRS agar (HiMedia Laboratories Pvt. Limited, India) was used for culturing Lactobacillus species in a veterinary microbiology laboratory (Mabna Lab, Karaj, Iran).

### Blood sampling

To collect blood samples from the jugular vein of the calves, 10 mL pre-evacuated tubes were used on day 3, 30, and 60, 3 h after the morning feeding. Two tubes were used for each calf to obtain serum (with no additive in the tube) and plasma samples (heparinized tubes with sodium fluoride and potassium oxalate). The blood samples were transported on ice to the farm adjacent laboratory and allowed to clot at room temperature for 30 min before serum was separated by centrifugation (3000 rpm for 15 min at 4 °C) for serum separation. Heparinized tubes were immediately centrifuged (3000 rpm for 15 min at 4 °C), and plasma samples were segregated. The serum and plasma samples were stored at − 20 °C until subsequent analysis.

Plasma samples were analyzed to determine glucose (intra and inter-assay CV of 1.07 and 1.86, respectively), cholesterol (intra and inter-assay CV of 0.94 and 1.42, respectively), blood urea nitrogen (BUN, intra and inter-assay CV of 2.18 and 2.76, respectively), albumin (intra and inter-assay CV of 0.78 and 1.11, respectively), and total protein (intra and inter-assay CV of 1.45 and 1.88, respectively) using commercially available diagnostic kits (Biorex-Fars, Shiraz, Iran) and a calibrated autoanalyzer (BT-1500, Biotecnica Instruments, Rome, Italy). The concentrations of inflammatory markers were measured in the serum of calves using ELISA kits and an ELISA reader (DANA 3200, Garny, Iran) as follows:

- Bovine serum tumor necrosis factor-alpha (TNF-α, Bioassay, Shanghai, China, Cat No: E0019Bo, intra and inter-assay CV of 1.53 and 4.18, respectively)
- Bovine serum amyloid A (SAA, Bioassay, Shanghai, China, Cat No: E0023Bo, intra and inter-assay CV of 2.18 and 6.32, respectively)
- Interferon-gamma (IFN-γ, Karmania Pars Gene, Kerman, Iran, intra and inter-assay CV of 2.18 and 2.76, respectively)
- Interleukin-6 (IL-6, Karmania Pars Gene, Kerman, Iran, intra and inter-assay CV of 1.43 and 3.24, respectively)

An immunoturbidimetric assay^22^ as performed to determine serum haptoglobin (Hp) concentration (anti-bovine immunoturbidimetric assay; Biorex-Fars, Shiraz, Iran, intra and inter-assay CV of 4.6 and 4.81, respectively) using an automated biochemical analyzer (BT-1500, Biotecnica Instruments, Rome, Italy). Malondialdehyde (MDA, Nalondi™-Lipid Peroxidation Assay Kit-MDA; Navand-salamat, Urmia, Iran) was also measured in serum samples using a plate reader (DANA 3200, Garny, Iran) at 550 nm.

Fatty acid profile of plasma samples was determined only from the samples obtained from 60-day-old calves. All the chemical solvents and reagents utilized in lipid extraction and preparation of the fatty acid methyl esters (FAME) were of analytical grade, and solvents were redistilled before use. As described in Folch et al.^23^ to avoid fatty acid oxidation, lipid extraction was carried out three times with chloroform/methanol (C/M, 2/1, v/v) to a final volume of 100 mL administered under the argon gas blanket. The flasks, after each extraction step, were centrifuged (1800 g for 10 min), and the organic fraction was separated and injected into a 100 mL volumetric flask. Afterward, they were treated with anhydrous Na-sulfate to be dry and then vaporized using a rotary evaporator (Büchi, Switzerland) at 40 °C under a vacuum. Using mild methanolysis/methylation via methanolic hydrochloric acid (HCl/MeOH), FAME were prepared by a method explained by Ichihara and Fukubayashi^24^. Hexane was utilized as a solvent to extract, and gas chromatography analysis was conducted after drying with anhydrous Na-sulfate. Nonadecanoic acid was utilized as an internal standard.

For FA analysis, Agilent 6890 gas chromatograph (Agilent Technologies, Santa Clara, California, United States) equipped with an autoinjector (Agilent 7683 series, Santa Clara, California, United States) and FID detector was used. Samples (1 µL) were injected in split mode, 50:1, into a RESTEK column for FAME (Rtx®-2330, 105 m × 250 µm × 0·2 µm; catalog No. 10729; serial No. 1525353, Restek Corporation, 110 Benner Circle, Bellefonte, PA16823). The detector and injector temperatures were set at 250 °C. The carrier gas was N2 at a flow of 1 mL/min. Based on the method described by Lee et al.^25^, the oven temperature was set at the gradient temperature to rise with some modifications. It was 70 °C for 1 min, and then was increased from 5 °C/min to 100 °C and was kept for 2 min. Then, the column temperature was increased from 10 °C/min to 175 °C and was maintained for 35 min. The temperature was increased from 4 °C/min to 225 °C and was kept for 35 min. Based on a FAME standard mix (GLC 463, Nu-Chek Prep Inc., Elysian, MN; reference mixture 47 885, Supelco Inc., Bellefonte PAGLC 463 reference mixture, http://www.nu-chekprep.com/10 11 catalog.pdf), individual peaks were identified.

### Statistical analysis

A standard deviation of 100 g ADG was assumed, with the minimum meaningful difference in ADG set at 65–75 g/d, based on the values published previously^26,27^. A power test analysis was run (α = 0.05 and power (1–β) = 0.80), and the expected sample size of 12 calves/treatment was achieved for growth performance, the most reliable parameter for power test determination^28,29^. Repeated measures variables (starter feed intake, DMI, FA intake, ADG, feed efficiency, skeletal growth parameters) within the same calf were analyzed with fixed effects of treatment, time (d), the interaction between treatment and time, and sex of calf, and random effect of calf nested within treatment.

To determine the most appropriate variance–covariance structure for our analysis, three variance–covariance structures (autoregressive type 1, compound symmetry, and Toeplitz) were tested, and an autoregressive type 1 covariance structure was selected as the best fit based on the Bayesian information criterion. All residuals were tested for normality using the Shapiro–Wilk statistic and the UNIVARIATE procedure of SAS (version 9.4, SAS Institute Inc., Cary, NC), and for homogeneity of variance using Levene's test, and visually assessed using quantile–quantile plots. Data (starter feed intake, DMI, ADG, BW, skeletal growth, and fecal scores) that did not meet the assumptions of normality of the residuals had to be log-transformed (base 10). After log transformation, the distribution of the data was retested, and the data were normally distributed.

The data for plasma metabolites and fecal microbial counts were analyzed using the above mixed-effects model without the effect of time. The initial values of body measures (BW and skeletal growth) were used as covariates for the corresponding variables. Data were reported as least squares mean, and the Tukey–Kramer adjustment was applied to account for multiple comparisons. The SLICE statement of the MIXED procedure (PROC MIXED) of SAS was used to conduct partitioned analyses of the LSM for interactions when required. The data for health and fecal score were analyzed by logistic regression using a binomial distribution in the GLIMMIX procedure in SAS. The significance threshold was set at *P* ≤ 0.05; trends were declared at 0.05 < *P* ≤ 0.10.

### Multivariate analyses

Multivariate statistical analyses of blood parameters of calves fed different diets were performed using the web-based metabolomics data processing tool MetaboAnalyst 5.0 (Pang et al.,^30^; http://www.metaboanalyst.ca for detailed methodology). Data were transformed using the generalized square root transformation and then scaled by Pareto (mean divided by the square root of the standard deviation of each variable) to correct for heteroscedasticity, reduce skewness, and mask effects. Principal component analysis (PCA) was applied to obtain an overview of blood parameters including albumin, cholesterol, glucose, haptoglobin, IFN-γ, IL-6, MDA, SAA, TNF-α, total protein, and urea according to treatment on d 30 and d 60. Identification of significant blood metabolites affected by the dietary treatments was performed using random forest classification. In this process, 500 trees were used and each node had seven predictors using MetaboAnalyst 5.0^30^.

## Results

### Intake and growth performance

**Figure 1:**
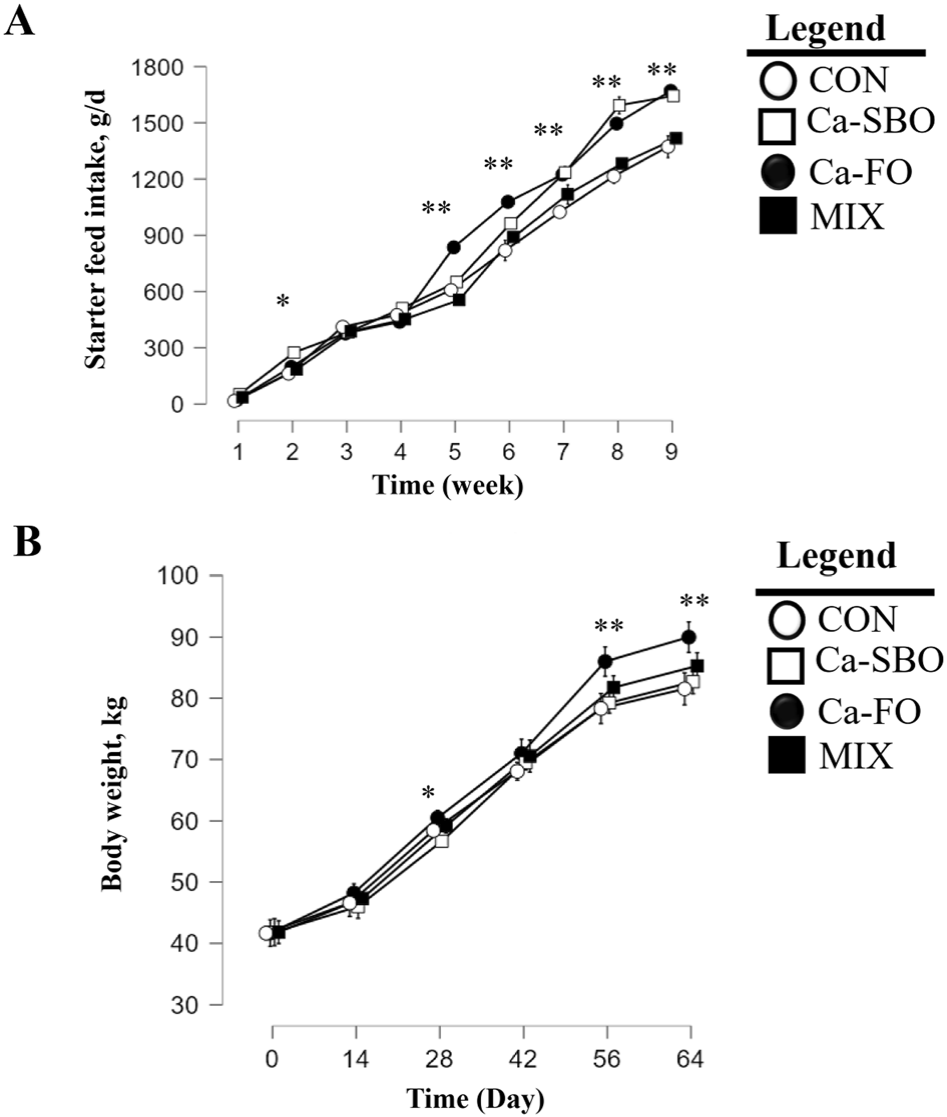
Effect of different fat sources on starter feed intake (**A**) and body weight (**B**) in dairy calves fed different diets. The four treatments included (1) a control group with no supplemented fat (CON), (2) a group fed 3% calcium salts of soybean oil (Ca-SBO), (3) a group fed 3% calcium salts of fish oil (Ca–FO), and (4) a group fed a mixture of 1.5% Ca-SBO and 1.5% Ca–FO on a dry matter basis (MIX). Symbols indicate a difference (*P = 0.01; **P < 0.01) between the groups at a given time. Data are presented as means ± SEM. The significance threshold was set at *P* ≤ 0.05; trends were declared at 0.05 < *P* ≤ 0.10.

Table 4 shows the results for starter feed intake, DMI, and FA intake from starter diets. Starter feed intake interacted with fat source and age. At week 2, calves fed Ca-SBO had greater starter feed intake than calves fed other feeds. At weeks 5 through 9, calves fed Ca–FO had greater starter feed than calves fed CON and MIX (Fig. 1A). Calves fed Ca-SBO and Ca–FO had greater starter feed intake and total DMI than calves fed MIX and CON throughout the experiment. Fat source and time interacted to affect the intake of FA from the starter diet at weeks 5 to 9 (Table 4). Calves fed Ca-SBO had the greater intake of C18:2 and C18:1, followed by the MIX and Ca–FO groups. Intake of C18:3 was greater in the Ca-SBO group, followed by the Ca–FO and MIX groups. The intake of C18:0 was greater in the Ca–FO group, followed by the MIX and Ca-SBO groups. Intake of EPA and DHA was greater in calves fed Ca–FO and MIX than the other diets. Fat source and age affected ADG (Table 5). Calves fed Ca–FO had higher ADG than the other treatments on d 28, 56, and 64. Calves fed Ca–FO had a significant increase in BW on d 28, 56, and 64 compared to the other treatments (Fig. 1B). The Ca–FO group showed greater feed efficiency throughout the experimental period (d 3—65), followed by the MIX, Ca-SBO and CON groups.

**Table 4:** Effects of different fat sources (calcium salts of soybean oil, fish oil or their mix) on starter and DMI intake and daily fatty acid intake in Holstein calves during cold season (n = 12 per treatment).

Measure	Treatments*				SEM	P-value^#^		
	CON	Ca-SBO	Ca–FO	MIX		F	T	F ˟ T
Starter feed intake, g/d (d 3 to 65)	677.8	811.4	815.1	702.6	24.83	< 0.01	< 0.01	< 0.01
Total DMI g/d (d 3 to 65)	1224.5	1358.1	1361.7	1249.2	25.53	0.01	< 0.01	< 0.01
Daily fatty acid intake from starter, g/d								
C16:0	2.46	5.68	6.82	5.81	0.046	< 0.01	< 0.01	< 0.01
C18:0	0.48	1.11	1.59	1.40	0.010	< 0.01	< 0.01	< 0.01
C18:1	4.56	10.30	10.15	8.82	0.072	< 0.01	< 0.01	< 0.01
C18:2	9.41	20.94	16.10	15.49	0.125	0.01	< 0.01	< 0.01
C18:3	0.53	1.11	1.07	0.99	0.007	0.01	< 0.01	< 0.01
EPA DPA, DHA	0	0	2.96	1.25	0.017	< 0.01	< 0.01	< 0.01

* Treatments were: CON: no supplemented fat source, Ca-SBO: calcium slats of soybean oil (3%, DM basis), Ca–FO: calcium slats of fish oil (3%, DM basis); MIX: a mixture of Ca-SBO and Ca–FO (1.5% each, DM basis).

^#^ F = effect of fat sources; T = effect of time; F × T = interaction between supplemental fat and time.

**Table 5:** Effects of different fat sources (calcium salts of soybean oil, fish oil, or their mix) on BW, average daily gain, body weight, feed efficiency, and skeletal growth (cm) in dairy calves during the cold season (n = 12 per treatment).

Item	Treatments*				SEM	*P*-values^#^		
	CON	Ca-SBO	Ca–FO	MIX		F	T	F × T
Body weight, kg								
Initial (d 3)	41.66	41.83	41.66	41.83	0.707	0.93	–	–
Weaning (d 65)	81.38^b^	82.37^b^	89.69^a^	85.07^b^	1.655	0.02	–	–
Average daily gain, kg/d (d 3 to 65)	0.64	0.65	0.77	0.70	0.019	0.03	< 0.01	< 0.01
Feed efficiency (d 3 to 65 )^2^	0.52	0.50	0.57	0.58	0.015	< 0.01	< 0.01	0.11
Withers height, cm	87.77	87.61	88.44	87.22	1.191	0.94	< 0.01	0.15
Body length, cm	70.27	70.72	69.88	69.66	0.903	0.85	< 0.01	0.32
Body girth, cm	88.38	89.50	89.50	88.94	1.345	0.92	< 0.01	0.06
Hip width, cm	23.05	22.83	23.11	23.77	0.431	0.47	< 0.01	0.34
Pin width, cm	9.15	9.19	9.91	9.11	0.304	0.14	< 0.01	0.17
Hip to Pin, cm	23.55	23.61	24.16	23.61	0.412	0.69	< 0.01	0.09

^a-c^ Means within a row with different superscripts differ (*P* < 0.05).

* Treatments were: CON: no supplemented fat source, Ca-SBO: calcium slats of soybean oil (3%, DM basis), Ca–FO: calcium slats of fish oil (3%, DM basis); MIX: a mixture of Ca-SBO and Ca–FO (1.5% each, DM basis).

^#^ F = effect of fat sources; T = effect of time; F × T = interaction between supplemental fat and time.

None of the structural growth indices, including withers height, body length, body girth, pin width, and hip width, were affected by fat supplementation during the experimental period.

### Blood FA profiles

**Figure 2:**
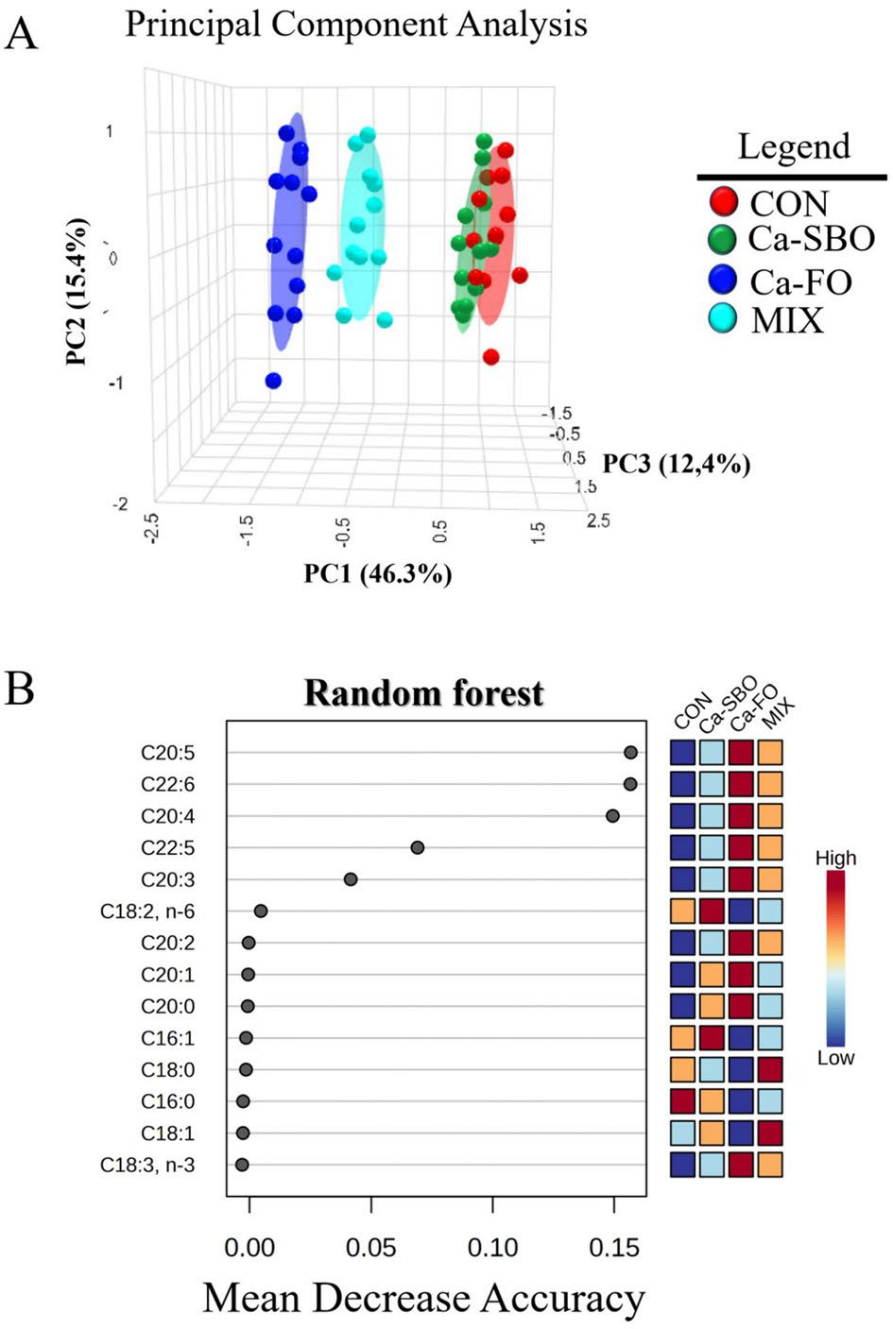
Principal component analysis (PCA) plot (**A**) and random forest (**B**) of serum fatty acid composition in dairy calves fed different diets. The four treatments included (1) a control group with no supplemented fat (CON), (2) a group fed 3% calcium salts of soybean oil (Ca-SBO), (3) a group fed 3% calcium salts of fish oil (Ca–FO), and (4) a group fed a mixture of 1.5% Ca-SBO and 1.5% Ca–FO on a dry matter basis (MIX).

Considering the results of the plasma FA profile of the calves at d 60 revealed that the concentration of linoleic acid was greater in calves fed the Ca-SBO diet compared with the other treatments (Table 6). Blood concentrations for long-chain n-3 polyunsaturated FA (PUFA) including EPA and DHA were greater in Ca–FO than in the other treatments, but dietary supplementation with fat did not affect plasma concentrations of linolenic acid (C18:3). Based on the serum FA composition, PCA analyses (Fig. 2A) showed a clear distinction between the dietary treatments on d 60. The first principal component (PC) accounted for 46.3% of the variance, whereas the second and third PCs contributed 15.4% and 12.4%, respectively. In addition, random forest analyses revealed that EPA, DHA, and arachidonic acid were the serum FA most affected by treatment diets, with the greater levels observed in calves fed Ca–FO than in the other treatments (Fig. 2B).

**Table 6:** Effects of different fat sources (calcium salts of soybean oil, fish oil, or their mix) on plasma concentration of selected fatty acid methyl esters in dairy calves during the cold season (d 60 of the experiment; n = 12 per treatment).

Item	Treatments*				SEM	*P*-value
	CON	Ca-SBO	Ca–FO	MIX		
C16:0	17.02	16.50	15.58	15.97	0.699	0.62
C16:1	0.616	0.626	0.572	0.564	0.032	0.46
C18:0	11.83	11.40	11.18	11.80	0.317	0.42
C18:1	10.43	10.61	10.49	10.94	0.541	0.91
C18:2, n-6	41.66^b^	43.09^a^	39.78^c^	40.24^c^	0.447	< 0.01
C18:3, n-3	0.642	0.654	0.684	0.678	0.031	0.76
C20:0	0.310^b^	0.338^ab^	0.350^a^	0.338^ab^	0.012	0.03
C20:1	0.776	0.846	0.874	0.806	0.037	0.08
C20:2	0.232^b^	0.244^b^	0.278^a^	0.258^ab^	0.010	0.04
C20:3	0.414^c^	0.554^b^	0.888^a^	0.794^a^	0.041	< 0.01
C20:4	0.400^c^	0.492^c^	1.520^a^	0.982^b^	0.045	< 0.01
C20:5	0.778^d^	0.876^c^	3.474^a^	2.325^b^	0.031	< 0.01
C22:5	0.444^c^	0.466^c^	0.794^a^	0.566^b^	0.018	< 0.01
C22:6	0.192^d^	0.254^c^	0.892^a^	0.606^b^	0.017	< 0.01

^a-c^ Means within a row with different superscripts differ (*P* < 0.05).

^a^ Treatments were: CON: no supplemented fat source, Ca-SBO: calcium slats of soybean oil (3%, DM basis), Ca–FO: calcium slats of fish oil (3%, DM basis); MIX: a mixture of Ca-SBO and Ca–FO (1.5% each, DM basis).

### Blood metabolites, and inflammatory markers

As shown in Table 7, calves fed the CON diet had significantly lower blood glucose concentrations than calves fed the other diets. Blood cholesterol concentrations were significantly greater in calves fed the Ca–FO diet than in calves fed the other diets. Fat supplementation significantly increased blood cholesterol concentrations compared to the CON diet. Calves fed the Ca-SBO diet had significantly lower total protein concentrations than calves fed the other diets.

**Table 7:** Effects of different fat sources (calcium salts of soybean oil, fish oil, or their mix) on blood metabolites, inflammatory cytokines, and oxidative markers in dairy calves during the cold season (n = 12 per treatment).

Item	Treatments#				SEM	*P*-value		
	CON	Ca-SBO	Ca–FO	MIX		F	T	F × T
Blood metabolites								
Glucose, mg/dL	77.62^b^	81.70^b^	89.95^a^	80.83^b^	1.76	< 0.01	< 0.01	0.32
Cholesterol, mg/dL	93.62^c^	100.0^b^	105.4^a^	102.0^ab^	1.50	< 0.01	< 0.01	< 0.01
Urea, mg/dL	15.07	15.70	15.24	15.65	0.58	0.83	< 0.01	0.85
Total protein, g/dL	5.44^bc^	5.27^c^	5.67^ab^	5.84^a^	0.10	< 0.01	0.52	< 0.01
Albumin, g/dL	2.98	2.92	3.11	3.08	0.06	0.16	< 0.01	0.52
Inflammatory markers								
TNF-α^4^, pg/mL	390.3^a^	358.7^b^	319.1^c^	355.5^b^	4.79	< 0.01	0.37	0.03
IFN-γ^5^, ng/mL	145.9^a^	141.3^a^	118.0^c^	129.9^b^	2.05	< 0.01	0.11	0.95
Haptoglobin, mg/dl	20.23^a^	18.32^b^	14.49^d^	16.67^c^	< 0.01	< 0.01	< 0.01	< 0.01
SAA^6^ mg/l	16.03^a^	13.60^b^	8.81^c^	13.17^b^	0.42	< 0.01	< 0.01	0.08
IL-6^7^ pg/mL	118.5^a^	111.6^b^	99.71^c^	107.6^b^	1.82	< 0.01	0.08	0.94
MDA^8^, nmol/ mL	18.98^a^	18.13^ab^	15.00^c^	16.99^b^	0.61	< 0.01	0.08	0.78

^a-c^ Means within a row with different superscripts differ (*P* < 0.05).

^a^ Treatments were: CON: no supplemented fat source, Ca-SBO: calcium slats of soybean oil (3%, DM basis), Ca–FO: calcium slats of fish oil (3%, DM basis); MIX: a mixture of Ca-SBO and Ca–FO (1.5% each, DM basis).

^4^ TNF-α = Tumor Necrosis Factor-alpha.

^5^ IFN-γ = *Interferon-gamma.*

^6^ SAA = Serum Amyloid A.

^7^ IL-6 = Interleukin-6.

^8^ MDA = Malondialdehyde.

**Figure 3:**
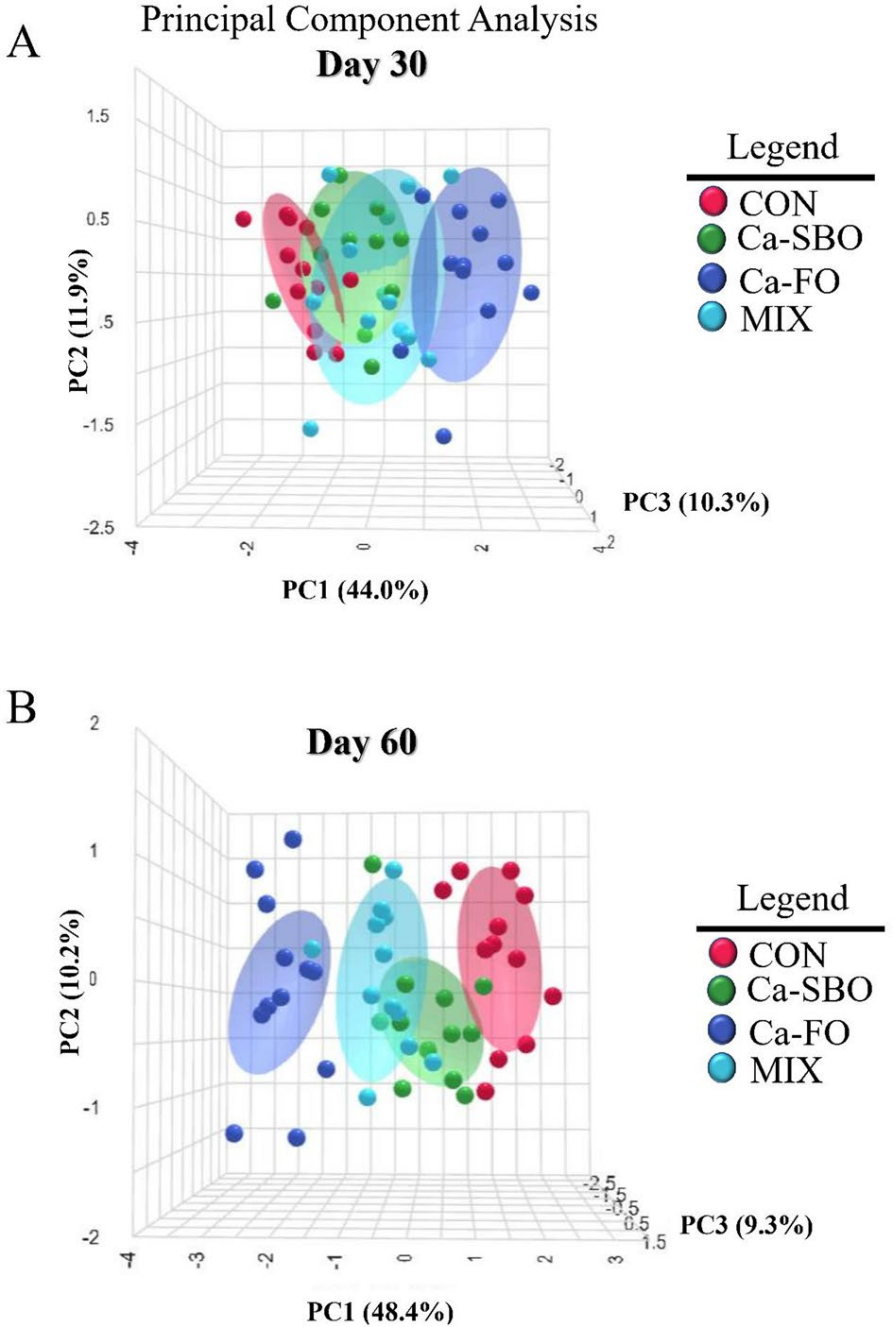
Principal component analysis (PCA) plot of blood metabolites, and inflammatory markers on d 30 (**A**) and d 60 (**B**) in Holstein calves fed different diets. The four treatments included (1) a control group with no supplemented fat (CON), (2) a group fed 3% calcium salts of soybean oil (Ca-SBO), (3) a group fed 3% calcium salts of fish oil (Ca–FO), and (4) a group fed a mixture of 1.5% Ca-SBO and 1.5% Ca–FO on a dry matter basis (MIX).

**Figure 4:**
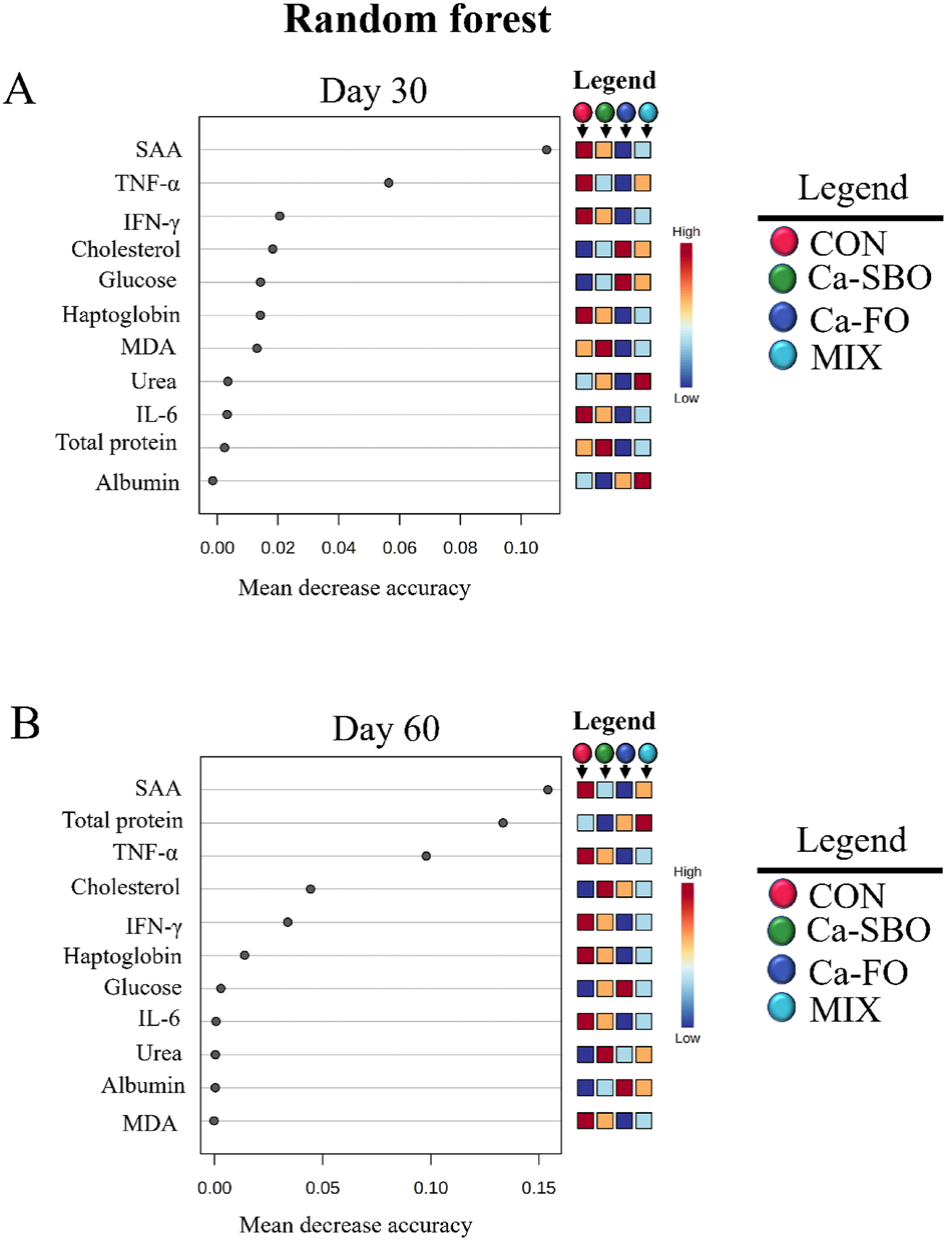
Random forest plot of blood metabolites, and inflammatory markers on d 30 (**A**) and d 60 (**B**) in Holstein calves fed different diets. The four treatments included (1) a control group with no supplemented fat (CON), (2) a group fed 3% calcium salts of soybean oil (Ca-SBO), (3) a group fed 3% calcium salts of fish oil (Ca–FO), and (4) a group fed a mixture of 1.5% Ca-SBO and 1.5% Ca–FO on a dry matter basis (MIX).

Inflammatory markers such as TNF-α, INF-γ, Hp, SAA, and IL-6 had the lower concentrations in the blood of calves fed Ca–FO compared with the other experimental treatments. In contrast, calves fed CON had the greater concentrations of TNF-α, Hp, and SAA in blood during the experimental period compared to calves fed the other treatment diets. Blood MDA concentrations (an indicator of oxidative stress) were lower in calves fed Ca–FO compared to calves fed the other treatment diets. The PCA analysis of blood metabolites and inflammatory markers showed that there were distinct differences between dietary treatments at d 30 and 60 of the study (Fig. 3A,B). At d 30 (Fig. 3A), the first PC explained 44% of the variance, and the second and third PC explained 11.9% and 10.3%, respectively (PC2 and PC3 = 22.2%). According to Fig. 3B, PC1 explained 48.4% of the variance, whereas PC2 and PC3 contributed 10.2% and 9.3%, respectively (PC2 and PC3 = 19.5%). Random forest analysis was used to identify changes in blood inflammatory markers in response to dietary treatment at d 30 (Fig. 4A) and d 60 (Fig. 4B). Results showed that SAA, TNF-α, and INF-γ were the most affected serum FA on d 30, whereas SAA, total protein, and TNF-α were the most affected on d 60, with calves fed Ca–FO having the lowest levels of inflammatory markers (Fig. 4A,B).

### Health indicators and fecal microbial counts

Table 8 summarizes the results of health indicators and fecal microbial counts. Supplementation with Ca–FO resulted in lower nasal discharge indices compared with the CON diet. The addition of Ca–FO and Ca-SBO to the starter diet resulted in the lower ear and eye scores compared with the CON diet, but the MIX diet did not improve health indices. In addition, supplementation of the starter feed with Ca–FO had a lower rectal temperature, ear skin temperature, and respiratory rate compared with calves fed the CON feed. It was also found that calves fed the CON feed had higher *E. coli* counts than calves fed the supplemental fat feed.

**Table 8:** Effects of different fat sources (calcium salts of soybean oil, fish oil, or their mix) on the fecal score, health indicators, and fecal microbial counts in dairy calves during the cold season (n = 12 per treatment).

Item (health indicators)	Treatments*				SEM	*P*-value		
	CON	Ca-SBO	Ca–FO	MIX		F	T	F × T
Fecal score^2^	0.144	0.088	0.072	0.088	0.03	0.37	0.17	0.76
Nasal discharge^3^	0.050^a^	0.040^ab^	0.030^b^	0.043^a^	0.00	0.04	0.13	0.96
Eye scores^4^	0.193^a^	0.160^b^	0.163^b^	0.183^a^	0.01	< 0.01	< 0.01	0.57
Ear scores^5^	0.513^a^	0.450^b^	0.453^b^	0.470^a^	0.01	< 0.01	< 0.01	0.59
Days with diarrhea	6.63^a^	3.15^b^	2.88^b^	3.12^b^	0.17	0.04	–	–
Rectal temperature, °C	38.92^a^	38.75^ab^	38.48^b^	38.71^ab^	0.15	0.03	–	–
Ear skin temperature, °C	36.42	36.11	35.53	35.97	0.74	0.08	–	–
Respiratory rate (times/min)	54.21	53.73	52.47	53.24	0.68	0.07	–	–
Respiratory clinical score	4	3	3	3	0.22	0.84	–	–
Fecal microbial counts (cfu/g)							–	–
*Lactobacillus*	5.12*10^7^	6.10*10^7^	6.66*10^7^	6.40*10^7^	0.30	0.06	–	–
*E. coli*	5.77*10^6^	4.44*10^6^	4.75*10^6^	5.08*10^6^	0.34	0.04	–	–
*Salmonella*	ND	ND	ND	ND	–	–	–	−

^a-c^ Means within a row with different superscripts differ (*P* < 0.05).

^a^ Treatments were: CON: no supplemented fat source, Ca-SBO: calcium slats of soybean oil (3%, DM basis), Ca–FO: calcium slats of fish oil (3%, DM basis); MIX: a mixture of Ca-SBO and Ca–FO (1.5% each, DM basis).

^2^ Feces score scale: 0 = firm feces, no diarrhea; 1 = soft feces, no diarrhea, 2 = mild diarrhea, and 3 = watery, severe diarrhea.

^3^ Nasal discharge (0 = Normal serous discharge; 1 = Small amount of unilateral cloudy discharge; 2 = Bilateral, cloudy or excessive mucus discharge and 3 = Copious bilateral mucopurulent discharge).

^4^ Eye scores (0 = Normal; 1 = Small amount of ocular discharge; 2 = Moderate amount of bilateral discharge and 3 = Heavy ocular discharge).

^5^ Ear scores (0 = Normal; 1 = Ear flick or head shake; 2 = Slight unilateral droop and 3 = Head tilt or bilateral droop).

## Discussion

The lower critical temperature for rearing calves is 15 °C^31^. In the current study, the ambient temperature was below this value, indicating persistent cold stress for the calves. Cold stress can occur in the first month of calf rearing below 10 °C, and temperatures below -2 °C cause cold stress until weaning. Calves in cold climates have increased metabolic rates and lower critical temperatures^32,33^, which has negative effects due to immature cold protection mechanisms and high energy requirements^34,35^. This stress results in lower calf performance and economic losses in the calf industry^36,37^. The nutritional needs of calves raised below their thermoneutral zone are difficult to meet because much of the feed energy is used for thermoregulation, leaving less energy for growth promotion^37,38^.

### Intake, growth performance, and blood FA profile

Young calves have only about 3% body fat reserves and therefore dietary energy supplementation is necessary to avoid the negative consequences of energy deficits caused by cold stress^3^. Supplemental fat may be beneficial for calves suffering from cold stress because it conserves glucose and AA for thermoregulation, which is critical when calves are exposed to temperatures below the thermal neutral zone^7^. In addition, extra fat can conserve energy that can be used for functions other than growth, including immune function^39^. However, there are limited data on the effects of supplemental fat on pre-weaned calves under cold stress. In the current study, calves given 3% Ca-SBO, Ca–FO, or a mix had higher starter feed intake compared with the CON group, indicating the potential advantage of high-fat diets in cold weather. Our data indicate that young calves increase their energy intake during cold stress, and feed intake is not limited by metabolic factors as fat supplements (3% of the stater diet) are fed. Holt (2014) reported that calves raised in winter months consumed more calf starter than did those born in other months. In dairy cows, fat supplements differ markedly in their effects on DMI.

Researchers have previously reported that chronic cold stress can activate various inflammatory mechanisms and eventually increase circulating inflammatory cytokines^40–42^. Although this is the first study to report the effects of inflammation on preweaning feed intake of dairy cows, Kuhla^43^ and Gouvêa et al.,^44^ have found in separate reviews that pro-inflammatory cytokines, hypothalamic inflammation, and stress-induced inflammation negatively affect feed intake. As shown in Table 7, supplementation with Ca–FO results in lower inflammatory indices compared with control and Ca-SBO or MIX treatments, which may be an effective mechanism to explain the higher dry matter intake (DMI) of calves supplemented with Ca–FO.

As expected, feeding Ca-SBO to pre-weaned calves resulted in increased intake of C18 (linoleic and linolenic acids) PUFA, whereas Ca–FO resulted in increased intake of long-chain n-3 PUFA (EPA, and DHA) compared with CON. Calf starter diets typically contain large amounts of corn grain and soybean meal, which have a high proportion of linoleic acid in their fat content as n-6 FA, resulting in a high n-6: n-3 ratio (approximately 15:1,^8^). However, feeding soybean oil did not change this ratio or have a positive effect on starter feed intake and the growth performance of dairy calves^45^. This negative effect was observed in dairy calves when starter feeds high in n-6 FA was ingested, possibly due to the pro-inflammatory effect of n-6 FA^46^. Researchers have reported that supplementation with various n-3 FA sources, such as milk replacers with fish oil and linseed oil^47^, can improve the intake and growth performance of calves before weaning. The results of our study suggest that cold weather increases animal appetite, resulting in a higher intake of starter feeds when supplemented with fat compared with low-fat feeds. In addition, our results suggest that n-3 FA may have a more favorable effect on the starter feed intake compared to n-6 FA during the cold season.

The current study showed that feeding Ca–FO resulted in higher ADG, feed efficiency, and final weight because the starter feed provided more energy and other nutrients for weight gain. Our results are consistent with previous studies reporting that feeding n-3 FA in the form of Ca salts from flaxseed oil in the calf starter before weaning improved weight gain and feed efficiency compared to control diets^48,49^. In addition, studies by Hill et al.^6^ and Kadkhoday et al.^50^ showed that calves fed linseed oil had higher feed efficiency compared with calves fed the control diet. Mohtashami et al.^13^ also found that feed efficiency was improved in calves fed Ca salts of fish oil compared to pre-weaned calves fed Ca salts of soybean oil under heat stress. Although little information is available on calves, at least some of the improved average daily gain (ADG) and feed efficiency observed in calves supplemented with Ca–FO may be related to their inflammatory status and the effects of systemic inflammation on growth hormone and the growth hormone-insulin-like growth factor-1 (IGF-1) axis^51,52^.

Dietary PUFA can alter several physiological processes, including plasma lipid levels, immune function, insulin action, and cell regulation^53^. As previous studies have shown^54–56^, neonates rapidly increase their ability to oxidize FA after birth. As a result, increased PUFA intake in the preweaning period may increase the ability to oxidize FA, promote gene expression for oxidation, and decrease esterification, leading to changes in body composition. A study by Melendez et al.^57^ compared the effect of n-3 FA in milk replacers with that of n-6 and n-9 FA in canola oil on serum FA profiles in preweaning calves. They found that calves fed fish oil had higher n-3 FA concentrations than calves fed canola oil, but the sum of n-6 and n-9 FA did not differ in serum between treatments. According to our results, the observed differences in plasma FA composition could be seen as reflecting differences in diet., as shown by the higher intake of C16:0, C18:0, C18:1, and C18:3 FA in calves fed Ca–FO and Ca-SBO compared with those fed CON, with the lowest intake of all FA except C18:2. In addition, the Ca-SBO diet increased in blood n-6 FA, while the Ca–FO and MIX diets increased DPA, EPA, and DHA levels, as expected.

As part of the analysis of blood FA data, both PCA plots and random forest analysis were used. According to the PCA plots, there was a clear separation between the different treatment groups, with significant differences in calves fed the Ca–FO diet and the MIX diet. In addition, random forest analysis showed that long-chain n-3 PUFA, including DPA, DHA, and 20:4 (n-6), were most affected by the diets. Calves fed the Ca–FO diet had the greater concentrations of PUFA than calves fed the other diets, which were strongly related to the fat content of the starter diet. These results suggest that the inclusion of n-3 PUFA in the diets of calves before weaning, particularly in the form of fish oil, can significantly alter the FA profile of their blood and increase the concentration of important long-chain n-3 PUFA.

### Blood parameters and inflammatory markers

Calves fed the CON diet had lower blood glucose concentrations than calves fed the other diets. The Ca–FO with supplementation of omega-3 FA had a better effect on increasing plasma glucose levels, which can be attributed to the increased intake of starter feed. Starter feed intake plays the main role in regulating blood glucose concentration in dairy calves^58^. Ghasemi et al.^12^ reported that calves fed SBO had higher glucose concentrations than calves without supplementation. It has been suggested that glucose can be saved by fat supplementation and used for other purposes^12^. In addition, previous studies have shown that supplementation with long-chain n-3 FA increases hepatic glycerol gluconeogenesis and thus positively stimulates hepatic glucose production in humans and laboratory animals^59,60^, which needs further investigation in ruminants. Blood cholesterol concentration was increased in calves fed fat supplemented starter diets compared with CON, which is consistent with previous studies on calves fed supplemental fat^12,61^. Thus, our results suggest that higher blood cholesterol concentrations in calves raised during the cold season may have a preventive effect against diarrhea. Further studies are needed to elucidate the mechanisms involved. The lowest TP was observed in calves fed Ca-SBO in the current study. This is likely due to the higher requirement for lipoprotein synthesis to transport absorbed lipids in the blood observed in calves fed high-fat starter diets^62^. It is worth noting that previous studies on dairy calves have recommended feeding high protein to improve nitrogen intake in calves fed soybean oil^45^.

The study found that calves fed Ca–FO had lower concentrations of inflammatory markers (TNF-α, INF-γ, Hp, SAA, and IL-6) and MDA (an indicator of oxidative stress) compared to other experimental treatments. This indicates that the inclusion of fish oil, with its anti-inflammatory effects and n-3 fatty acids, benefits calves raised in cold preweaning conditions. On the other hand, the CON diet resulted in higher concentrations of TNF-α, Hp, and SAA, suggesting the pro-inflammatory properties of this particular diet in the study. The relevant literature supports these findings by highlighting the numerous metabolic activities of the long-chain FA present in fish oil that contribute to their anti-inflammatory effects^63^. The mechanisms involved in the regulation of inflammatory effects are not fully understood, but evidence suggests that long-chain FA activates cellular communication through the synthesis of prostaglandins, TNF-α, and IFN-γ, and influences other factors such as nitric oxide^64^. The lowest MDA level as an indicator of oxidative stress was found in the Ca–FO diet compared with other diets, suggesting that PUFA in fish oil calcium salts is not susceptible to oxidation during the cold season. While the specific mechanisms behind these effects need further investigation, the results provide substantial evidence for the superiority of Ca–FO in terms of its beneficial effects on inflammation and oxidative stress in such cold conditions. Breakdown of the dietary lipids to free fatty acids occurs rapidly and, in fact, more rapidly than the biohydrogenation process. Thus, large amounts of unsaturated oils can “overwhelm” the biohydrogenation process and result in undesirable effects on the rumen microbial population. During the biohydrogenation process, intermediate compounds with trans-double bonds are produced. One of these is conjugated linoleic acid (CLA), which has received much attention from the medical community because of its potent anti-cancer effects and other health benefits.

### Health indicators and fecal microbial counts

Calves receiving Ca–FO had more favorable fecal scores and fewer days with diarrhea than other experimental treatments, partly due to the effects of n-3 FA on animal health. When comparing FA in the starter feeds, results showed that n-6 FA was less effective in improving fecal scores than n-3 FA. Previous studies indicated that feeding soybean oil, which is rich in n-6 FA, to young calves may increase fecal fluidity and the animals showed more dietary diarrhea during the experimental period^11^. In contrast, Kadkhoday et al.^50^ indicated that milk-fed calves fed n-3 FA-rich flaxseed oil in the starter diet had better fecal scores during the study. In the study conducted by Garcia et al.^49^, diarrhea severity showed a linear tendency to decrease with increasing essential FA intake.

Lower rectal and ear skin temperatures in calves fed Ca–FO in starter feed indicated better health indices when calves received this FA supplement. Previous studies showed lower rectal temperature when calves were fed linseed oil, which is rich in n-3 FA^65^. This may be mainly related to the pro-inflammatory effect of n-6 FA (linoleic acid) and the anti-inflammatory effect of n-3 FA^6,9,49^. In this study, the number of *E. coli* was higher in calves fed CON forage. This suggests that feeding high-fat diets in the cold season makes calves less susceptible to pathogens, regardless of the FA source.

These results highlight the importance of selecting appropriate fat sources and providing optimal levels of essential FA in calf diets to promote health and growth. Further research is needed to elucidate the mechanisms underlying these effects and to optimize nutritional strategies for rearing dairy calves.

## Conclusions

The study showed that the addition of fat to starter feed can improve the performance of dairy calves before weaning under cold conditions. However, the type of fat used affected growth performance and inflammatory status. Specifically, supplementation with Ca–FO resulted in significant improvements in ADG, BW, feed efficiency, and fecal scores. In contrast, CON had higher levels of IL-6, haptoglobin, TNF-α, and SAA, along with lower blood glucose and cholesterol levels. These results suggest that supplementation with n-3 FA (Ca–FO) provides greater benefits in promoting growth and reducing inflammation in dairy calves exposed to cold conditions compared to n-6 FA (Ca-SBO).

## Data Availability

The datasets used and/or analyzed during the current study are available from the corresponding author on reasonable request.
